# Use of a Cytobrush for Sampling the Ear Canal of Dogs With Otitis Externa

**DOI:** 10.1111/vde.70036

**Published:** 2025-11-10

**Authors:** Nicoly Radaeli Atanasio, Gabriela Reis Ledur, Danilo Marcondes Filho, Daniel Guimarães Gerardi

**Affiliations:** ^1^ Veterinary Clinics Hospital Federal University of Rio Grande Do Sul Porto Alegre Brazil; ^2^ Institute of Mathematics and Statistics Federal University of Rio Grande Do Sul Porto Alegre Brazil

**Keywords:** dog, exfoliative cytological investigation, swab diagnostic techniques

## Abstract

**Background:**

Cytological examination of the ear canal is essential for evaluating dogs with otitis externa (OE). The conventional sampling method uses a swab. However, the cytobrush (gynaecological cervical brush), already used for cytological collection from other anatomical sites, has not been adequately investigated for this purpose in dogs with OE.

**Objectives:**

To compare the cytobrush as a sampling tool for the ear canal of dogs with OE and compare it with the swab technique.

**Animals:**

Thirty ears from 17 dogs with OE, presented at a veterinary teaching hospital, were included for sampling.

**Materials and Methods:**

Cytological samples were collected using both a cytobrush and a swab in random order. Two independent and blinded evaluators quantified micro‐organisms (cocci, bacilli, yeasts), mononuclear cells, polymorphonuclear cells and epithelial cells. Animal discomfort during sampling was assessed using a scoring system.

**Results:**

No significant differences were found between the methods regarding the presence of micro‐organisms or inflammatory and epithelial cells (*p* > 0.05), indicating equivalence between techniques. The intraclass correlation coefficient (ICC > 0.9) demonstrated high reproducibility between evaluators. Although the oto‐podal reflex was more frequent with the cytobrush, it did not significantly impact overall animal discomfort.

**Conclusions and Clinical Relevance:**

The cytobrush is an effective, safe and well‐tolerated sampling method, and may be considered a viable alternative to the swab for collecting samples from the ear canal of dogs with OE.

## Graphical abstract

Cytological examination of the ear canal is essential for evaluating dogs with otitis externa (OE). The conventional sampling method uses a swab. However, the cytobrush (gynaecological cervical brush), already used for cytological collection from other anatomical sites, has not been adequately investigated for this purpose in dogs with OE. To compare the cytobrush as a sampling tool for the ear canal of dogs with OE and compare it with the swab technique. The cytobrush is an effective, safe and well‐tolerated sampling method, and may be considered as a viable alternative to the swab for collecting samples from the ear canal of dogs with OE.

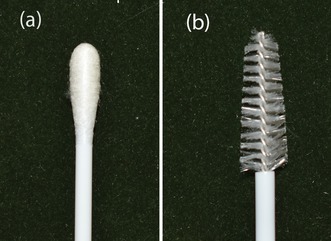

## ZUSAMMENFASSUNG

**Hintergrund:**

Eine zytologische Untersuchung des Ohrkanals ist bei Hunden mit einer Otitis externa (OE) für eine Evaluierung essenziell. Bei der konventionellen Probenahme Methode wird ein Wattestäbchen eingesetzt. Die Zytobrush (eine gynäkologische Zervixbürste), die bereits zur zytologischen Probenahme von anderen anatomischen Körperstellen eingesetzt wird, ist für diesen Einsatz bei Hunden mit OE noch nicht ausreichend untersucht worden.

**Ziele:**

Ein Vergleich der Zytobrush als Probenahme Instrument für den Ohrkanal des Hundes mit OE und der Probenahme mittels Tupfer.

**Tiere:**

Dreißig Ohren von 17 Hunden mit OE, die in einer veterinärmedizinischen Klinik vorgestellt worden sind, wurden zur Probennahme inkludiert.

**Materialien und Methoden:**

Es wurden zytologische Proben mittels sowohl Zytobrush wie auch einem Tupfer in zufälliger Reihenfolge entnommen. Zwei unabhängige und geblindete UntersucherInnen quantifizierten die Mikroorganismen (Kokken, Bazillen, Hefen), mononukleäre Zellen, polymorphonukleäre Zellen und Epithelzellen. Es wurde das Unbehagen der Tiere während der Probenahme mit einem Bewertungssystem erfasst.

**Ergebnisse:**

Es konnten keine signifikanten Unterschiede zwischen den Methoden in Bezug auf das Auftreten von Mikroorganismen oder Entzündungs‐ und Epithelzellen (*p* > 0,05) gefunden werden, was eine Gleichwertigkeit der Methoden zeigt. Der Intraklassen Korrelations‐Koeffizient (ICC > 0,9) zeigte eine hohe Reproduzierbarkeit zwischen den UntersucherInnen. Obwohl der Ohr‐Pedal Reflex bei der Zytobrush häufiger ausgelöst wurde, hatte dies keinen signifikanten Einfluss auf das allgemeine Wohlbefinden des Tieres.

**Schlussfolgerungen und klinische Bedeutung:**

Die Zytobrush Technik ist eine effektive, sichere und gut tolerierte Probenahme Methode und kann als praktikable Alternative zur Probenahme mittels Tupfer aus dem Ohrkanal bei Hunden mit OE eingesetzt werden.

## 摘要

**背景:**

对外耳炎(OE)患犬进行耳道细胞学检查是评估病情的重要步骤。传统的取样方式为棉拭子。然而，细胞刷(妇科宫颈刷)已被用于其他解剖部位的细胞采集，但其在犬外耳炎耳道中的应用尚缺乏充分研究。

**目的:**

比较细胞刷与棉拭子在犬外耳炎耳道细胞学取样中的表现。

**动物:**

纳入来自某兽医教学医院的17只外耳炎患犬，共30个耳道。

**材料与方法:**

使用细胞刷和棉拭子分别以随机顺序在同一耳道中采集样本。由两名独立且盲法的评估者对微生物(球菌、杆菌、酵母菌)、单核细胞、多形核细胞及上皮细胞进行计数。动物在取样过程中的不适程度通过评分系统进行评估。

**结果:**

两种取样方法在微生物、炎症细胞和上皮细胞的检出方面无显著差异(p > 0.05)，提示两者效果等同。组内相关系数(ICC > 0.9)显示评估者之间具有高度一致性。尽管细胞刷更易引发耳–足反射，但对整体不适度无显著影响。

**结论与临床意义:**

细胞刷是一种有效、安全且耐受性良好的耳道细胞学取样方法，可作为棉拭子的可行替代方案，用于犬外耳炎耳道样本采集。

## Résumé

**Contexte:**

L‘examen cytologique du conduit auditif est essentiel pour évaluer les chiens atteints d‘otite externe (OE). La méthode d‘échantillonnage conventionnelle utilise un écouvillon. Cependant, la cytobrosse (brosse gynécologique cervicale), déjà utilisée pour le prélèvement cytologique sur d‘autres sites anatomiques, n‘a pas été suffisamment étudiée à cette fin chez les chiens atteints d‘OE.

**Objectifs:**

Comparer le cytobrush en tant qu‘outil de prélèvement pour le conduit auditif des chiens atteints d‘OE et le comparer à la technique du coton‐tige.

**Animaux:**

Trente oreilles provenant de 17 chiens atteints d‘OE, présentés dans un hôpital universitaire vétérinaire, ont été incluses dans l‘échantillonnage.

**Matériels et méthodes:**

Les échantillons cytologiques ont été prélevés à l‘aide d‘une cytobrosse et d‘un écouvillon dans un ordre aléatoire. Deux évaluateurs indépendants et en aveugle ont quantifié les micro‐organismes (coques, bacilles, levures), les cellules mononucléaires, les cellules polymorphonucléaires et les cellules épithéliales. L‘inconfort des animaux pendant le prélèvement a été évalué à l‘aide d‘un système de notation.

**Résultats:**

Aucune différence significative n‘a été observée entre les deux méthodes en ce qui concerne la présence de micro‐organismes ou de cellules inflammatoires et épithéliales (p > 0,05), ce qui indique une équivalence entre les deux techniques. Le coefficient de corrélation intraclasse (CCI > 0,9) a démontré une reproductibilité élevée entre les évaluateurs. Bien que le réflexe oto‐podal ait été plus fréquent avec la cytobrosse, cela n‘a pas eu d‘impact significatif sur l‘inconfort global des animaux.

**Conclusions et pertinence clinique:**

La cytobrosse est une méthode de prélèvement efficace, sûre et bien tolérée, qui peut être considérée comme une alternative viable au coton‐tige pour le prélèvement d‘échantillons dans le conduit auditif des chiens atteints d‘OE.

## 要約

**背景:**

外耳炎(OE)の犬を評価するには、外耳道の細胞診が不可欠である。従来のサンプリング法ではスワブが用いられている。一方、他の解剖学的部位からの細胞診採取に既に使用されているサイトブラシ(婦人科子宮頸部用ブラシ)は、OEの犬においてこの目的で十分に検討されていない。

**目的:**

本研究の目的は、OE犬の外耳道サンプリングツールとしてのサイトブラシおよびスワブ法を比較検討することであった。

**対象動物:**

獣医教育病院を受診したOE犬17頭の30耳を検体採取に用いた。

**材料と方法:**

細胞診検体は、サイトブラシとスワブを用いてランダムな順序で採取した。2名の独立した盲検化評価者が、微生物(球菌、桿菌、酵母)、単核細胞、多形核細胞、および上皮細胞を定量した。採取中の動物の不快感は、スコアリングシステムを用いて評価した。

**結果:**

微生物、炎症細胞、上皮細胞の存在に関して、各方法間に有意差は認められなかった(p > 0.05)。これは、各方法間の同等性を示している。クラス内相関係数(ICC > 0.9)は、評価者間で高い再現性を示した。サイトブラシを用いた場合、耳足反射の頻度は高かったものの、動物の全体的な不快感には有意な影響を与えなかった。

**結論と臨床的意義:**

サイトブラシは、効果的で安全かつ忍容性の高い採取法であり、外耳道炎(OE)の犬の外耳道から検体を採取する際に、スワブに代わる有効な選択肢となり得た。

## Resumo

**Contexto:**

O exame citológico do conduto auditivo é essencial para a avaliação de cães com otite externa (OE). O método convencional de coleta utiliza um swab. No entanto, a escova citológica cytobrush (escova cervical ginecológica), já utilizada para coleta citológica de outros sítios anatômicos, não foi adequadamente investigada para esse fim em cães com OE.

**Objetivos:**

Comparar a cytobrush como ferramenta de coleta de amostras do conduto auditivo de cães com OE com a técnica de swab.

**Animais:**

Trinta orelhas de 17 cães com OE, atendidos em um hospital veterinário universitário, foram incluídas para coleta de amostras.

**Materiais e Métodos:**

Amostras citológicas foram coletadas utilizando‐se tanto a cytobrush quanto o swab, em ordem aleatória. Dois avaliadores independentes e cegos quantificaram microrganismos (cocos, bacilos, leveduras), células mononucleares, células polimorfonucleares e células epiteliais. O desconforto dos animais durante a coleta foi avaliado utilizando‐se um sistema de pontuação.

**Resultados:**

Não foram encontradas diferenças significativas entre os métodos quanto à presença de microrganismos ou células inflamatórias e epiteliais (*p* > 0,05), indicando equivalência entre as técnicas. O coeficiente de correlação intraclasse (CCI > 0,9) demonstrou alta reprodutibilidade entre os avaliadores. Embora o reflexo otopodal tenha sido mais frequente com a cytobrush, isto não impactou significativamente o desconforto geral do animal.

**Conclusões e Relevância Clínica:**

A cytobrush é um método de amostragem eficaz, seguro e bem tolerado, podendo ser considerada uma alternativa viável ao swab para coleta de amostras do conduto auditivo de cães com otite externa.

## RESUMEN

**Introducción:**

El examen citológico del conducto auditivo externo es esencial para evaluar a perros con otitis externa (OE). El método convencional de muestreo utiliza un hisopo. Sin embargo, el cepillo citológico (cepillo cervical ginecológico), ya utilizado para la toma de muestras citológicas de otras localizaciones anatómicas, no se ha investigado suficientemente para este fin en perros con OE.

**Objetivos:**

Comparar el cepillo citológico como herramienta de muestreo del conducto auditivo externo de perros con la técnica del hisopo.

**Animales:**

Se incluyeron para el muestreo treinta oídos de 17 perros con OE, atendidos en un hospital veterinario universitario.

**Materiales y métodos:**

Se recogieron muestras citológicas utilizando tanto un cepillo citológico como un hisopo en orden aleatorio. Dos evaluadores independientes y con valoración ciega cuantificaron microorganismos (cocos, bacilos, levaduras), células mononucleares, células polimorfonucleares y células epiteliales. El malestar de los animales durante el muestreo se evaluó mediante un sistema de puntuación.

**Resultados:**

No se encontraron diferencias significativas entre los métodos con respecto a la presencia de microorganismos o células inflamatorias y epiteliales (*p* > 0,05), lo que indica equivalencia entre las técnicas. El coeficiente de correlación intraclase (ICC > 0,9) demostró una alta reproducibilidad entre evaluadores. Si bien el reflejo otopodal fue más frecuente con el citobrush, no influyó significativamente en la incomodidad general del animal.

**Conclusiones y relevancia clínica:**

El citobrush es un método de muestreo eficaz, seguro y bien tolerado, y puede considerarse una alternativa viable al hisopo para la toma de muestras del conducto auditivo de perros con otitis externa.

## 1 Introduction

Otitis externa (OE) is a highly prevalent condition in dogs, affecting approximately 15%–20% of the dogs presented to veterinary practice [1]. This inflammation of the external ear canal may have a wide range of primary and secondary causes, and is often associated with predisposing and perpetuating factors [2]. These elements frequently interact, resulting in clinical presentations that can vary from mild acute episodes to severe, chronic and recurrent cases [1, 3].

As a consequence of the complex aetiology of OE, its diagnosis and the identification of all contributing factors are challenging, and require precise and reliable methods to assess the severity of the disease and guide appropriate treatment [1, 4, 5]. Among the available diagnostic tools, cytological evaluation of the ear canal stands out as an accessible, rapid and indispensable technique for identifying microbial morphology (such as coccoid or rod‐shaped bacteria and yeasts), inflammatory and epithelial cells [4, 5, 6].

Cytological sampling is traditionally performed using cotton‐tipped swabs, a well‐established technique that is simple to execute and generally effective and inexpensive [1, 4, 5, 6]. However, alternative methods have been explored, including the use of urethral catheters to collect material from deeper regions of the ear canal [7]. Cytobrushes, commonly used in medical and veterinary contexts, have shown superior sampling capabilities, particularly in veterinary ophthalmology [8, 9] and in human gynaecological cytological investigation [10, 11]. These brushes are composed of nylon bristles that, when rotated or gently rubbed against epithelial surfaces, promote exfoliation and retention of cells. In dogs, brushes also have been used to obtain cytological samples from the respiratory tract [12, 13, 14, 15, 16], conjunctiva [9] and gastrointestinal tract [17]. Such evidence supports their potential as a sampling tool for the canine ear canal.

Despite this, no systematic studies have evaluated the comparative effectiveness of cytobrushes versus cotton swabs in the diagnosis of canine OE. The present study was therefore designed to prospectively and blindly assess whether the cytobrush is a viable and well‐tolerated alternative to the conventional swab for otic cytological investigation. Specifically, we aimed to compare microbial and cellular representativeness between techniques, including coccoid and rod‐shaped bacteria, yeasts, inflammatory infiltrates (mononuclear and polymorphonuclear cells) and epithelial cells, as well as to evaluate animal discomfort during sample collection. We hypothesised that the cytobrush would be a viable and equally or more effective method than the cotton swab for obtaining representative samples from the ear canal of dogs with OE, and that the cytobrush sampling procedure would be as well‐tolerated as the traditional swab.

## 2 Materials and Methods

### 2.1 Ethical Considerations

This study was conducted at Federal University of Rio Grande do Sul (UFRGS, Brazil), following approval of the study protocol by the Institutional Animal Care and Use Committee (IACUC) of UFRGS (approval no. 46456). Informed consent was obtained from all owners before inclusion through the signing of a written consent form.

### 2.2 Animals

This was a prospective, randomised, blinded study including dogs diagnosed with OE. All dogs were recruited from the clinical caseload of the Veterinary Clinical Hospital at UFRGS School of Veterinary Medicine.

Dogs were included if they presented with a clinical history and signs consistent with OE, such as head shaking, otic pruritus, pain, otorrhea, erythema of the concave pinna and external auditory meatus, hyperplasia and/or oedema. Each dog underwent a physical examination, otoscopic inspection and cytological evaluation of the external ear canal. In cases of bilateral otitis, each ear was assessed and included independently. Dogs were enrolled regardless of age, sex, reproductive status, breed or the primary cause of the otological condition. Dogs were excluded if, within the 4 weeks before inclusion or at the time of enrollment, they had received ear cleaners, ceruminolytic/ceruminosolvent products or topical or systemic medications for the treatment of OE.

### 2.3 Sample Collection Techniques and Slide Preparation

Cytological samples were collected from each ear using both a cotton‐tipped swab and a cytobrush. The order of sampling was randomised for each ear by a simple coin toss to ensure unbiased sequencing.

**FIGURE 1:**
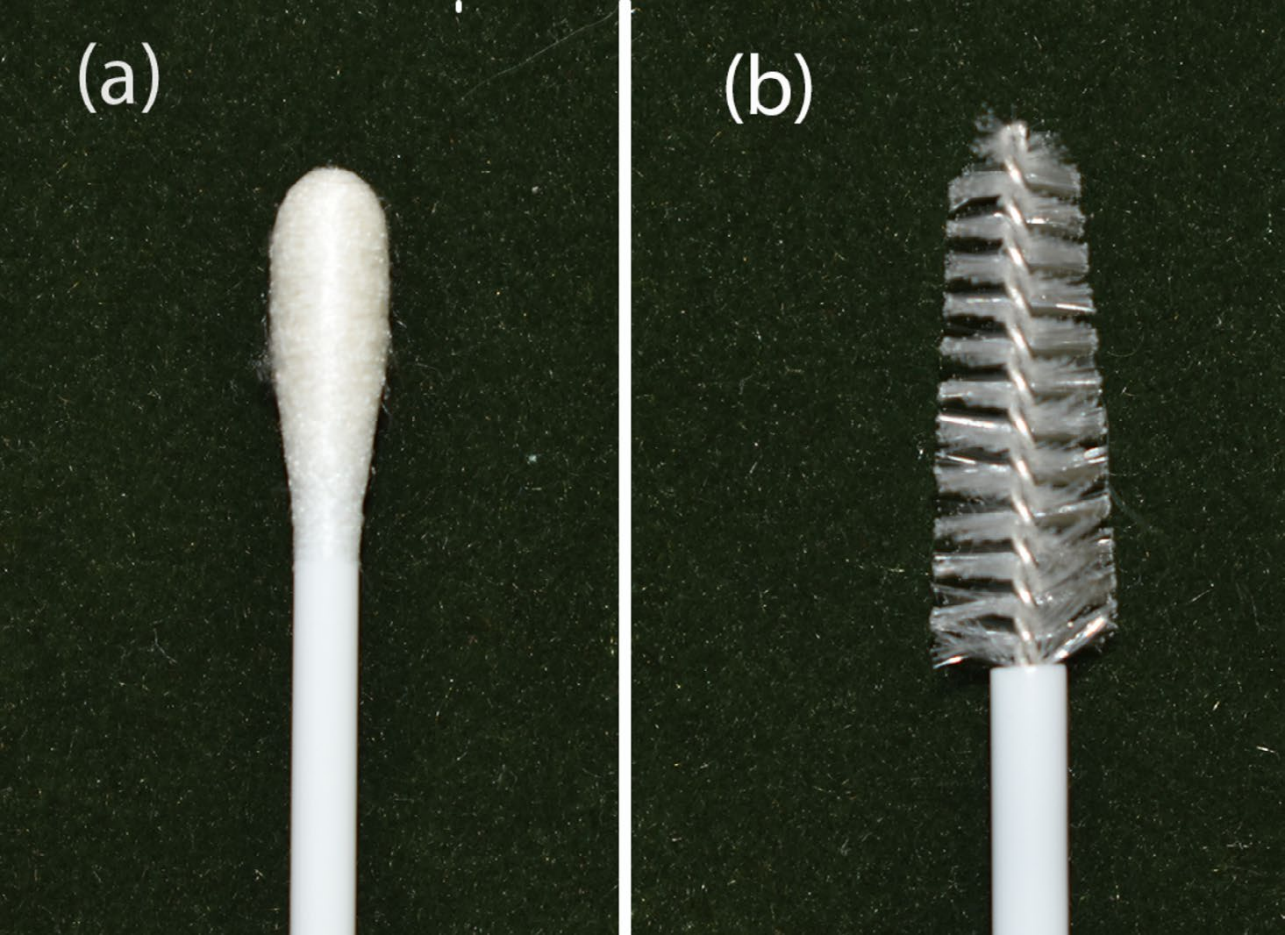
Instruments used for cytological sampling of the external ear canal in dogs with otitis externa. (a) Cotton swab and (b) gynaecological cervical cytobrush.

For the swab technique, a sterile cotton‐tipped applicator with a plastic shaft (15 mm length, model K41‐0201B, Olen; Figure 1a) was inserted into the lumen of the ear canal until the end of the vertical canal and rotated clockwise three times (360°). The swab tip was then rolled once along the longitudinal axis of a microscope slide to transfer the sample. The cytobrush technique followed the same guidelines yet used a sterile gynaecological cervical brush (ABSORVE) composed of a 180 mm plastic shaft with a 20 mm nylon bristle brush at the tip (Figure 1b).

All slides were stained using a Romanowsky‐type rapid stain (Panoptic; NEWPROV). Fixation was intentionally omitted to avoid removal of the lipid content. Therefore, slides were analysed and photographed immediately after preparation.

### 2.4 Cytological Evaluation

All slides were initially examined by a single investigator (N.R.A). Slides were first screened at low magnification (×40 or ×100) to identify areas with visible cellularity. One region was then selected for high‐power evaluation (×1000, oil immersion). Selection criteria included the presence of inflammatory cells, nuclear debris and micro‐organisms; in their absence, areas containing keratinocytes or generalised basophilic debris were chosen. Regions with excessive overlapping were excluded to minimise errors in structure identification [7, 18]. Each selected ×1000 oil immersion field was photographed using ZEN 2.3 software (Carl Zeiss). After assessing one region, scanning at low power was resumed in a continuous horizontal movement, from the groove to the opposite edge, until a total of 10 non‐overlapping fields had been captured [18].

The microphotographs were subsequently analysed independently by two blinded investigators (D.G.G and G.R.L). The number of yeasts, cocci, rods, polymorphonuclear and mononuclear cells, and epithelial cells was counted. The final score for each category was obtained by calculating the mean count across the 10 fields [7, 19]. A maximum of 30 micro‐organisms and 20 cells per field was counted, as established in previous studies [7, 18].

### 2.5 Assessment of Discomfort

In order to evaluate the discomfort shown by dogs during sampling, behavioural responses were recorded from the moment the swab or brush was introduced into the ear canal until its complete removal. A numeric scoring system was developed specifically for this study. Five behaviours indicative of discomfort—vocalisation, head shaking, attempt to bite the examiner, restlessness requiring physical restraint and the presence of an oto‐podal reflex—were scored individually, with one point assigned for each behaviour observed. Thus, the total discomfort score for each procedure ranged from 0 (no signs of discomfort) to 5 (all signs present). The overall discomfort score for each technique was calculated by summing the individual scores from all ears.

### 2.6 Statistical Methods

The sample size determination considered a significance level of 5% and an 82% probability of detecting moderate differences between the two methods with respect to the population means of the variables analysed [20].

Inter‐rater agreement between the two evaluators was calculated using the intraclass correlation coefficient (ICC). Agreement levels were interpreted as follows: < 0.5 = poor, 0.5–0.75 = moderate, 0.75–0.9 = good and > 0.9 = excellent [21].

The Wilcoxon signed‐rank test was applied to assess statistically significant differences between the two sampling techniques for each cytological finding and discomfort score. A *p*‐value < 0.05 was considered significant. All analyses were performed using the open‐source software R.

## 3 Results

### 3.1 Animals

Of the 17 dogs included in the study, 13 had bilateral otitis and four had unilateral otitis, totaling 30 sampled ears. Six male and 11 female dogs were included with a mean age of 8.1 years, range 2–13 years (Table 1).

**TABLE 1:** Animal ID, ear ID, breed, age, sex and additional characteristics of the dogs included in the study.

Animal inclusion number	Ear inclusion number	Breed	Age (years)	Sex	Side affected
1	1 & 2	Dachshund	2	M	B
2	3 & 4	German Shepherd dog	7	F	B
3	5 & 6	Basset hound	9	M	B
4	7 & 8	Shih tzu	8	F	B
5	9	Shih tzu	9	F	U
6	10	Lhasa apso	13	F	U
7	11	Shih tzu	10	M	U
8	12 & 13	Golden retriever	9	F	B
9	14 & 15	Shih tzu	8	F	B
10	16 & 17	Shih tzu	8	F	B
11	18 & 19	Beagle	10	M	B
12	20 & 21	Mixed‐breed	13	F	B
13	22 & 23	Mixed‐breed	4	F	B
14	24 & 25	Pinscher	10	F	B
15	26	Mixed‐breed	13	M	U
16	27 & 28	Mixed‐breed	3	M	B
17	29 & 30	German spitz	3	F	B

Abbreviations: B, bilateral; F, female; M, male; U, unilateral.

### 3.2 Comparison Between Collection Techniques

No statistically significant differences were observed between the cytobrush and swab methods for any of the cytological findings assessed (*p* > 0.05), indicating that both methods were equivalent (Table 2).

**TABLE 2:** Comparison of cytological findings from the ear canals of dogs with otitis externa using swab (S) and cytobrush (B) collection techniques.

	Method	Median	Min.	Max.	*p*
Cocci	S	0.375	0.00	30.00	0.4455
	B	0.250	0.00	29.40	
Bacilli	S	4.5850	0.00	30.00	~1
	B	4.693	0.00	30.00	
*Malassezia* spp.	S	11.650	0.00	29.90	0.1747
	B	12.775	0.00	30.00	
Mononuclear cells	S	0.02	0.00	0.60	0.3711
	B	0.05167	0.00	0.85	
Polymorphonuclear cells	S	0.0	0.00	0.60	0.1814
	B	0.0	0.00	18.85	
Epithelial cells	S	1.625	0.00	6.20	0.8693
	B	1.750	0.00	6.40	

### 3.3 Inter‐Rater Reliability

A high degree of similarity between results from the two collection methods was obtained by the two evaluators for the quantification of cocci, rods, *Malassezia* spp., polymorphonuclear cells and epithelial cells (Table 3). The ICC values ranged from 0.93 to 0.997, indicating excellent agreement levels. However, for mononuclear cells, the agreement was perfect with the swab method (ICC = 1), yet only moderate with the cytobrush (ICC = 0.527).

**TABLE 3:** Intraclass correlation coefficients (ICC) demonstrating inter‐rater reliability for the quantification of cytological findings using swab (S) and cytobrush (B) techniques for ear canal sampling.

	Method	ICC	IC 95% (ICC)
Cocci	S	0.99	0.980 < ICC < 0.995
	B	0.982	0.963 < ICC < 0.991
Bacilli	S	0.997	0.994 < ICC < 0.999
	B	0.994	0.986 < ICC < 0.997
*Malassezia* spp.	S	0.997	0.987 < ICC < 0.999
	B	0.997	0.981 < ICC < 0.999
Mononuclear cells	S	1	—
	B	0.527	0.214 < ICC < 0.742
Polymorphonuclear cells	S	0.946	0.891 < ICC < 0.974
	B	0.995	0.990 < ICC < 0.998
Epithelial cells	S	0.93	0.859 < ICC < 0.966
	B	0.978	0.954 < ICC < 0.989

### 3.4 Discomfort Assessment

No significant differences were found in discomfort scores between the swab and cytobrush techniques (*p* = 0.1096) (Table 4).

**TABLE 4:** Number of sampling events in which each behavioural reaction occurred, comparing swab and cytobrush techniques for ear canal sampling.

Collection method	Head movement	Bite attempt	Vocalisation	Oto‐podal reflex	Restlessness	Total score	*p*
Swab	15	1	14	05	7	42	0.1096
Brush	15	1	14	10	7	47	

## 4 Discussion

In our study, although not statistically significant, there was a trend toward higher median epithelial cell counts in samples collected with the cytobrush compared to swabs. Previous studies have shown better recovery of epithelial cells with cytobrushes than with cotton swabs in human Pap smear tests [11] and feline conjunctival cytological investigation [22], which is likely to be a consequence of the greater exfoliation caused by brush bristles [23]. However, because the auditory canal is not lined with mucosa, unlike the uterus or conjunctiva, epithelial exfoliation may be limited, explaining the absence of a significant difference between techniques.

Likewise, nonsignificant trends toward higher counts of bacilli, *Malassezia* and inflammatory cells with the brush may be related to the clinical presentation of OE and sampling depth. A previous study comparing urethral catheter and swab collection in dogs with OE also found no significant differences, except for polymorphonuclear cells, which were more numerous in catheter samples owing to deeper collection within the horizontal canal in suppurative otitis [7]. In our study, both instruments were inserted only up to the junction between the vertical and horizontal canals, which may explain the absence of differences.

The ICC between evaluators was excellent for most variables with both methods, contrasting with previous studies on ear and skin cytological investigation that reported ICC values ranging from moderate to good [7, 18, 24]. The high level of agreement observed is likely to have resulted from the use of digitised images, which ensured that both observers examined identical microscopic fields, thereby reducing variability. A previous study comparing the evaluation of whole slides with digitised images also demonstrated greater reproducibility with the latter [25]. Additionally, the adoption of standardised counting limits of 30 micro‐organisms [7, 18, 24] and 20 cells [7, 18] per field, as described in the literature, probably minimised subjectivity and contributed to the overall reliability.

The high ICC values (0.93–0.997) indicate excellent agreement between evaluators for cocci, rods, *Malassezia*, polymorphonuclear cells and epithelial cells. By contrast, the lower ICC for mononuclear cells in brush samples (0.527) and the perfect agreement with swabs (1.0) were likely to have been a consequence of the rarity of this cell type, identified in only two of the 30 ears examined, which limited data representativeness and reduced agreement [18, 19].

During sampling, the cytobrush occasionally became entangled in the hair of dogs with dense coats, making removal more difficult, particularly for less experienced clinicians, owing to hair traction. However, discomfort scores were similar between instruments. Sample collection was generally well tolerated, with minimal behavioural discomfort observed for both methods. The pinnal–pedal reflex occurred more frequently with the brush, possibly owing to greater friction of its bristles against the epithelium, inducing a pruritic sensation. No additional discomfort or trauma was observed immediately after sampling with either method, except in one dog with suppurative otitis, which is likely to have been related to the combined manipulation.

Although both methods showed similar cytological representativeness and tolerance, some distinctions were observed. The brush proved more adaptable in stenotic ear canals, probably owing to the flexibility of its bristles, which conform to the lumen, and appeared to remove greater amounts of dense cerumen, while it may be more traumatic in suppurative otitis. Future studies should compare sampling performance according to the clinical form of OE.

Given the comparable diagnostic information and minimal discomfort, the routine replacement of cotton swabs by cytobrushes may not be justified, especially considering their low cost and wide availability. However, patient anatomy, clinician experience and training context may influence instrument choice. From an environmental standpoint, both are single‐use devices with similar ecological impact and should be discarded as infectious waste. A limitation of this study was the lack of correlation between cytological findings and clinical severity (OTIS‐3 [0–3 Otitis Index Score] scoring), which could have provided a more comprehensive assessment, particularly for inflammatory cells which were observed at low frequency in this study.

The results of this study indicate that the cytobrush is a viable instrument for collecting cytological samples from the ear canal of dogs with OE. Microbial and cellular representativeness, as well as comfort during sampling, were similar to those obtained with the traditional cotton swab method. Future studies considering different clinical presentations of OE may provide further insights into the applicability of the cytobrush, contributing to the optimisation of otic cytological techniques in veterinary patients.

## Author Contributions

**Nicoly Radaeli Atanasio:** investigation (lead), writing – original draft, writing – review and editing. **Danilo Marcondes Filho:** formal analysis, methodology (supporting), investigation, writing – review and editing. **Gabriela Reis Ledur:** investigation (equal), writing – original draft, writing – review and editing. **Daniel Guimarães Gerardi:** conceptualization (lead), methodology (lead), investigation (equal), writing – review and editing, supervision.

## Conflicts of Interest

The authors declare no conflicts of interest.

## Data Availability

The data that support the findings of this study are available from the corresponding author upon reasonable request.
